# Health Co-Benefits of Green Building Design Strategies and Community Resilience to Urban Flooding: A Systematic Review of the Evidence

**DOI:** 10.3390/ijerph14121519

**Published:** 2017-12-06

**Authors:** Adele Houghton, Carlos Castillo-Salgado

**Affiliations:** 1 Biositu, LLC, 505D W Alabama St, Houston, TX 77006, USA; 2 Department of Epidemiology, Johns Hopkins Bloomberg School of Public Health, 615 N Wolfe St, Baltimore, MD 21205, USA; ccastil3@jhu.edu

**Keywords:** urban flood-related hazards, sustainable design, climate change mitigation, climate change adaptation, sustainable communities

## Abstract

Climate change is increasingly exacerbating existing population health hazards, as well as resulting in new negative health effects. Flooding is one particularly deadly example of its amplifying and expanding effect on public health. This systematic review considered evidence linking green building strategies in the Leadership in Energy and Environmental Design^®^ (LEED) Rating System with the potential to reduce negative health outcomes following exposure to urban flooding events. Queries evaluated links between LEED credit requirements and risk of exposure to urban flooding, environmental determinants of health, co-benefits to public health outcomes, and co-benefits to built environment outcomes. Public health co-benefits to leveraging green building design to enhance flooding resilience included: improving the interface between humans and wildlife and reducing the risk of waterborne disease, flood-related morbidity and mortality, and psychological harm. We conclude that collaborations among the public health, climate change, civil society, and green building sectors to enhance community resilience to urban flooding could benefit population health.

## 1. Introduction

Climate change is increasingly exacerbating existing population health hazards, as well as resulting in new negative health effects [1]. Flooding illustrates this amplifying and expanding effect. It has been the second leading cause of death from extreme weather events in the USA for many decades (averaging 81 mortalities per year from 1986 to 2015) [2]. In a local example, over the three-year period of 2015–2017, the Houston metropolitan area has sustained one 500-year flood event each year [3], culminating with Hurricane Harvey in August 2017. Preliminary reports estimate over 50 inches of rain falling on Houston and roughly 70 deaths throughout the state before Harvey dissipated [4]. While climate models are uncertain about whether climate change is increasing the frequency of hurricanes like Harvey, they show warming temperatures, increasing the intensity of extreme precipitation events (and, therefore, flooding) in many regions of the United States [1].

The direct health effects of flooding are injuries and drowning, which often are the result of individuals attempting to drive or walk through or near flooded areas [5,6,7,8]. The indirect health effects of flooding include: exacerbated respiratory diseases such as asthma caused by compromised indoor air quality in previously flooded buildings [9,10,11,12,13,14,15,16,17]; risk of increased incidence of mosquito-borne diseases several weeks after heavy precipitation events if land use configurations support larval development, particularly if the event ends a dry spell [18,19,20,21]; waterborne diseases and infections of the eye, ear, nose, throat, or skin caused by compromised water quality following flooding events [22,23]; and, mental health concerns associated with the loss of loved ones, population displacement, loss of property, and economic hardship [5,22,23].

Given the interplay between the built environment and flooding-related injuries and deaths, architectural design has the potential to act as a protective climate change and health intervention by enhancing community resilience. For the purposes of this article, we have defined community resilience to align with its definition in the 2016 report published by the U.S. Global Change Research Program, *The Impacts of Climate Change on Human Health in the United States*: “… the ability to prepare and plan for, absorb, recover from, and more successfully adapt to adverse events” ([24], p. 30). In other words, resilience encompasses built environment interventions that plan for those events and, therefore, may attempt to reduce exposure. Also, exposure to flood waters may happen during the extreme weather event. It is not confined to the period of time after the rain stops.

This article and a companion literature review assess the state of the evidence linking green building strategies in the U.S. Green Building Council’s (USGBC) Leadership in Energy and Environmental Design^®^ (LEED) rating systems with the potential to reduce negative health outcomes following exposure to two climatic events. This article focuses on urban flooding events. The companion article, Associations between green building design strategies and community health resilience to extreme heat events: a systematic review of the evidence, addresses extreme heat events.

### 1.1. Green Building Practices and Human Health

LEED is the preeminent voluntary, third party-verified green building metric used in the USA and internationally. An average of 1.5 million square feet (sq. ft.) of construction space is certified as LEED projects daily [25]. New commercial and residential buildings, existing commercial buildings, and neighborhood development projects can be certified under the family of rating systems by meeting both the minimum requirements of mandatory “prerequisites” and forty percent of available voluntary credits. Higher levels of certification are awarded to projects meeting 50% (Silver), 60% (Gold), and 80% (Platinum) of available points [26].

In Versions 1–3 of the rating systems governing new commercial construction, prerequisites and credits are organized into six categories: Sustainable Sites, Water Efficiency, Energy & Atmosphere, Materials & Resources, Indoor Environmental Quality, and Innovation [26]. LEED prerequisites and credits outline measurable requirements for improving a building or development’s environmental performance. The overwhelming majority of LEED credits focus on reducing energy use, water use, and/or greenhouse gas emissions. Only five LEED credits (Sustainable Sites Credit 5.1: Site Development—Protect or Restore Habitat, Sustainable Sites Credit 5.2: Site Development—Maximize Open Space, Sustainable Sites Credit 6.1: Stormwater Design—Quantity Control, Sustainable Sites Credit 6.2: Stormwater Design—Quality Control, Water Efficiency Credit 1: Water Efficient Landscaping) could be said to directly reduce building occupant exposure to flood waters by increasing pervious surface or treating stormwater effluent. None of them ask project teams to incorporate climate scenarios into the assessment process to take into account projected future changes in precipitation amount or intensity [27].

Most LEED requirements are performance-based [26]. In other words, rather than requiring a specific roof construction to reduce the adverse effects of the urban heat island, the requirement (in this case, Sustainable Sites Credit 7.2: Heat Island Effect—Roof) lays out a minimum performance standard using the solar reflectance index, a measure of a surface’s ability to reflect solar heat. It is therefore not possible to predict with accuracy which design and construction solutions were employed by a LEED certified project based on a review of LEED credit achievement. Instead, it is more appropriate to consider LEED credit achievement as an indicator of a site or neighborhood’s potential resilience to climate change.

A study carried out by USGBC found that, while health-related issues are mentioned throughout the LEED rating systems, they are not applied in a systematic, evidence-based manner [28]. Only a handful of prerequisites and credits explicitly safeguard the health of construction workers and building occupants (Table 1). These requirements reference industry standards for design and construction practices protecting the construction job site and the completed building from dust accumulation, mold growth, insufficient ventilation, and absorption of volatile organic compounds emitted by liquid-applied products. All LEED rating systems for commercial construction also limit tobacco use after completion of construction [26]. The LEED rating systems specific to health care projects and secondary schools include additional health-related voluntary credits, such as providing outdoor places of respite at hospitals for use by patients and staff and designing appropriate acoustic environments in both hospitals and school classrooms [26,29]. Furthermore, Gray (2011)’s [30] assessment of the occupational health benefits associated with LEED credits in the commercial LEED rating systems concluded that health care staff satisfaction with views, space, and building layout were associated with perceptions of safety, security, and patient safety. Two reports linking specific green building strategies to the public health literature were produced as part of the development process for LEED for Neighborhood Development, the only LEED rating system addressing sustainable development at a larger scale than the individual building [31,32]. However, none of these studies addresses the possible links between green building strategies and the health effects of climatic events such as flooding.

### 1.2. Green Building Practices and Climate Change

Nearly half of all available points under the 2009 LEED rating system for New Construction and Major Renovations are designed to mitigate climate change (i.e., reduce greenhouse gas emissions), primarily by rewarding efforts to enhance energy efficiency, promote renewable energy, install environmentally sensitive refrigeration equipment, and reduce single-occupancy vehicle usage [33]. The green building industry’s focus on climate change mitigation has only recently been expanded to consider how they might be leveraged as a catalyst for reducing climate change vulnerability. For example, International Council for Local Environmental Initiatives (ICLEI), Local Governments for Sustainability (http://www.icleiusa.org), launched a companion challenge to the U.S. Conference of Mayors Climate Protection Agreement [34] at the Rio + 20 summit in June 2012 called the “Global Initiative on Urban Resilience” [35]. And, in September 2012, the American Institute of Architects launched a “Ten-Year Commitment to Make Design a Catalyst for Public Health” at the Clinton Global Initiative Conference, with three focus areas: public health, environmental sustainability, and resiliency to natural disasters [36].

Two previous studies have identified links between LEED credits and built environment resilience. Larsen et al. [27] propose so-called “No Regrets” and “Resilient” strategies that, if incorporated into design, construction, and operations and maintenance projects would reduce the risk of adverse impacts to a building’s structure and functionality after a climate change-related event. Pyke et al. [37], outline two climate-focused indices that assign values to a subset of LEED credits. The Climate Sensitivity Index assigns relative value to credits based on their reliance on climatic assumptions that are projected to shift as a result of climate change. The Climate Adaptation Opportunity Index assigns a relative value to the potential for a LEED credit to be achieved in such a way that reduces sensitivity and enhances the building’s adaptive capacity to changing climatic conditions. Neither study addresses population vulnerability to climatic events, particularly in relation to the localized vulnerability of a project site to the health and environmental effects of climatic events.

The study outlined in this article fills this research gap by performing a structured literature review to identify associations between green building practices and: (1) their impact on the risk of exposure to urban flooding; (2) related environmental determinants of health; (3) contributions to public health outcomes; and (4) contributions to built environment outcomes.

## 2. Materials and Methods

### 2.1. Conceptual Framework

**Figure 1:**
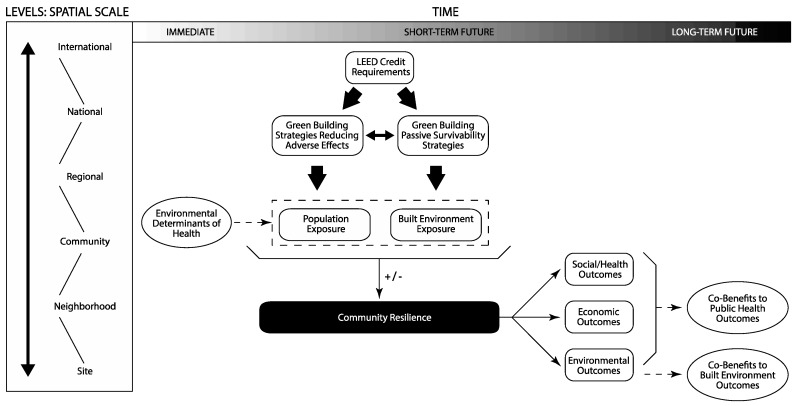
Conceptual framework: establishing an evidence base for associations between LEED credit requirements and climate change resilience outcomes.

The project adapted the social determinants of health conceptual framework [38,39,40] to establish a pathway for linking LEED with public health outcomes associated with climate change (Figure 1). LEED credit requirements can result in health outcomes affecting a variety of spatial scales, from the building level to the international level, and ranging in time from immediate to the long-term future. The reason for the breadth of LEED’s influence is the variety of strategies included in the rating system, which touch on many aspects of building design and operations.

The literature review used LEED credit requirements to assess the state of the evidence linking green building strategies with the potential to reduce the adverse effects of climate change with enhanced community resilience by mediating the impact of environmental determinants of health on population exposure to flooding events. Similarly, it assessed the potential for green building strategies designed to allow a building to continue to function during utility outages (also known as “passive survivability” [41]) to enhance community resilience by reducing built environment exposure to flooding events. The social, health, and economic outcomes after exposure to an event can result in co-benefits for public health. Related environmental outcomes can lead to co-benefits to the built environment.

### 2.2. LEED Credit Inclusion Criteria

**Figure 2:**
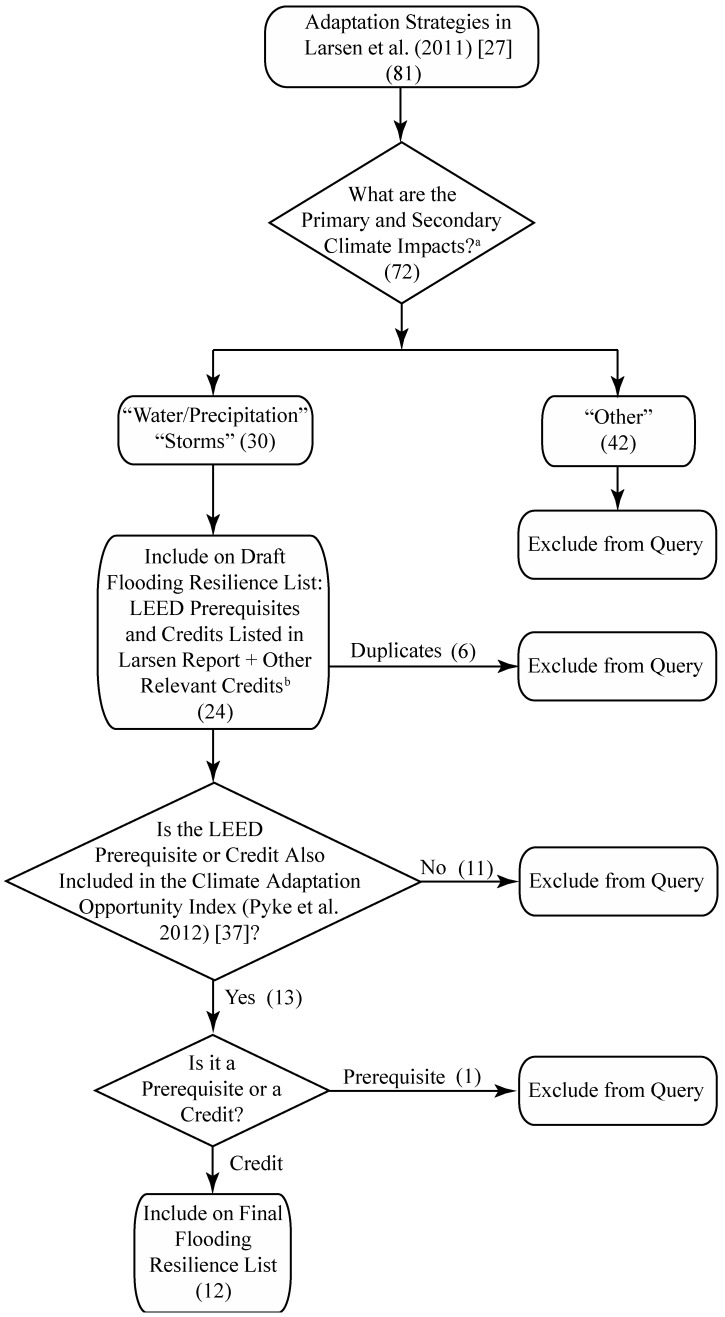
Flow chart of LEED credit inclusion criteria. ^a^ One or more primary or secondary impact may be designated under each adaptation strategy. ^b^ LEED prerequisites and credits may be listed under more than one adaptation strategy. Additionally, LEED for New Construction credits that are relevant to a strategy but not listed in the report were included in the assessment. PRISMA Flow Diagram adapted from Moher et al. (2009) [42].

The literature review assessed the relationship between green building strategies and urban flooding. Following Preferred Reporting Items for Systematic Reviews and Meta-Analyses (PRISMA) guidelines (Figure 2 and Figure A2), the review started with 81 “No Regrets” and “Resilient” strategies identified by Larsen et al. [27], 30 LEED prerequisites and credits identifying “Water/Precipitation” or “Storms” as either a primary or secondary climate impact were included in the initial Flooding Resilience list (Figure 2). The Larsen report lists some LEED prerequisites and credits under multiple “No Regrets” and “Resilient” strategies. Therefore, it was necessary to remove duplicates. Furthermore, credits that were relevant to an adaptation strategy but not included in the Larsen report were added to the list, resulting in a draft list of 24 LEED prerequisites and credits. A second round of screening was then applied. Eleven prerequisites and credits were excluded for not also appearing in the Climate Adaptation Opportunity Index developed by Pyke et al. [37], yielding a list of 13 prerequisites and credits. One prerequisite was removed, because all LEED-certified projects are required to achieve prerequisites; the literature review was tasked with identifying voluntary credits that could be prioritized by design teams to enhance a building project’s contribution to public health and built environment resilience to flooding events. Twelve LEED credits were included in the final flooding resilience literature review.

### 2.3. Literature Review Inclusion Criteria

**Figure A1:**
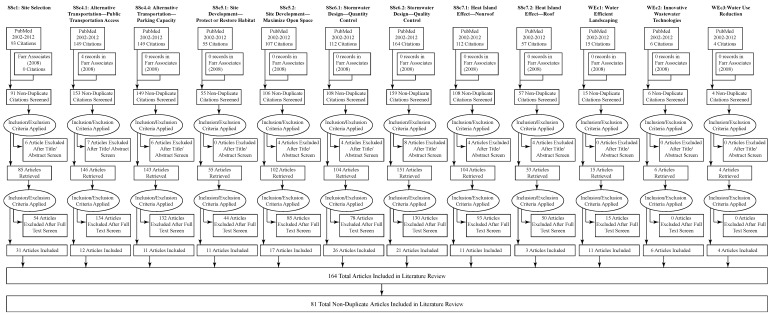
Flow Chart of Urban Flooding Literature Review Credit Inclusion Criteria. Note: PRISMA Flow Diagram adapted from Moher et al. (2009) [42].

**Figure A2:**
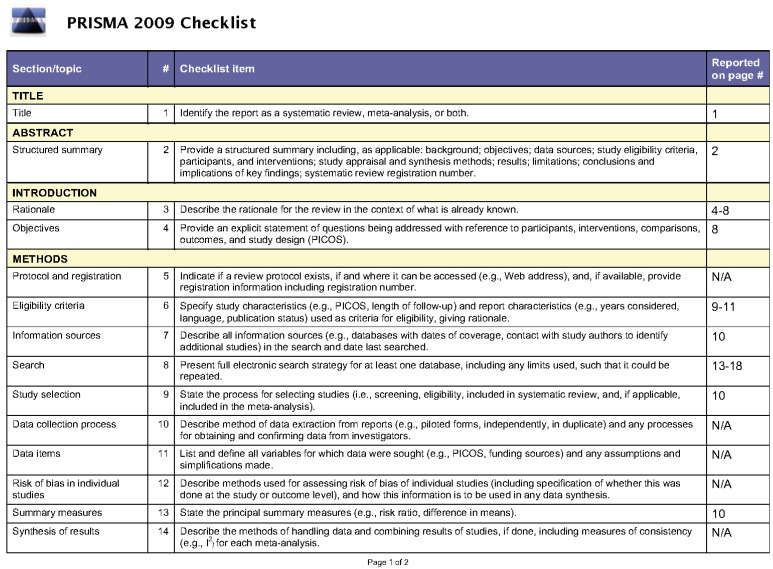

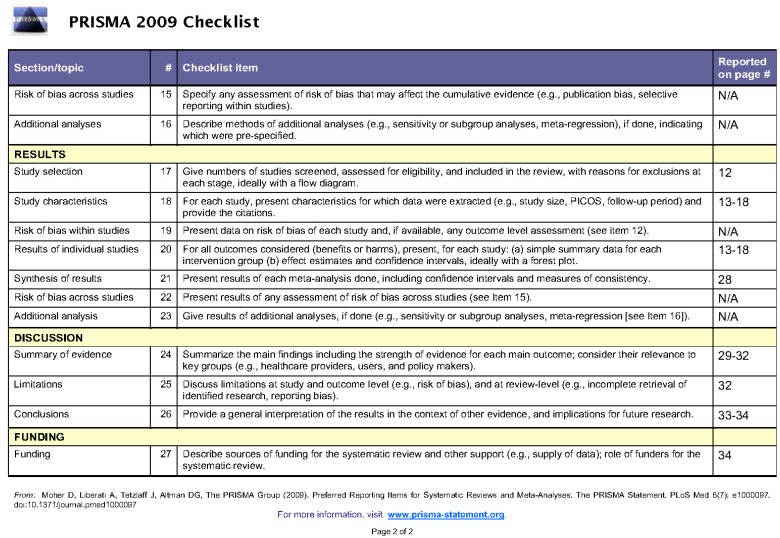
PRISMA Checklist.

The structured literature review first compared the LEED credits on the flooding resilience list with a 2008 review of the evidence supporting public health co-benefits associated with the LEED for Neighborhood Development rating system [32]. Green building strategies associated with each Credit on the Flooding Resilience list were then translated into National Library of Medicine Medical Subject Headings (MeSH^®^) [43]. Terms and submitted as queries via PubMed. For simplicity’s sake, MeSH terms have been listed in Table 3 without outlining specific combinations. See Supplemental Materials Table S1 for a table outlining the query combinations for specific LEED credits. Duplicate citations were removed from the review. Only articles in English from 2002–2012 were included in the full-text analysis. The full text of articles not excluded after a title/abstract screen were reviewed for evidence of links between LEED credit requirements and their potential impact on: risk of exposure to flooding, environmental determinants of health, co-benefits to public health outcomes, and co-benefits to built environment outcomes. See Figure A1 for a flow chart illustrating the literature review inclusion analysis for each LEED credit under review.

## 3. Results

Of the 12 LEED credits included in the flooding resilience literature review (Table 2), nine were drawn from the Sustainable Sites (SS) category and three were drawn from the Water Efficiency (WE) category. Notably, seven credits in the Sustainable Sites category overlapped with the heat resilience literature review (see companion article): SSc1: Site Selection, SSc5.1: Site Development—Protect or Restore Habitat, SSc5.2: Site Development—Maximize Open Space, SSc6.1: Stormwater Design—Quantity Control, SSc6.2: Stormwater Design—Quality Control, SSc7.1: Heat Island Effect—Nonroof, and SSc7.2: Heat Island Effect—Roof.

Flooding resilience queries for the 12 LEED credits under consideration returned 1027 results, 164 of which were relevant to the literature review, and 81 of which were non-duplicative (Figure A1). However, it should be noted that multiple reviews of a single article does not necessarily indicate duplicative results, because each review assessed links with a specific LEED Credit.

Sustainable Sites Credit 1: Site Selection requires projects to avoid development in or adjacent to prime farmland, endangered species habitat, parkland, floodplains, wetlands, and water bodies (Table 3). A literature review was conducted for the following topic areas: prime farmland, floodplains, endangered species, wetlands, water bodies, and parkland.

Prime farmland queried “Agriculture”, “Climate Change”, “Facility Design and Construction”, and “Urbanization” (17 citations were returned, five of which were relevant to the inquiry [44,45,46,47,48]). Design strategies meeting the credit requirements were found to reduce the risk of exposure to flooding events by influencing land use decisions, not contributing to sprawl, and not contributing to habitat fragmentation. By positively affecting the associated environmental determinants of health (access to opportunities for exercise, independence from automobiles, food and nutrition security, food safety, and contiguous habitat), these practices were found to reduce the risk of flooding-related injury, under- and mal-nutrition, infectious disease, and interaction between wildlife and humans. The co-benefits to built environment outcomes were identified as reducing development in areas without services; and, increasing access to local, productive agricultural land.

Floodplains queried “Floods” and “Facility Design and Construction” (seven citations were returned, five of which were relevant to the inquiry [49,50,51,52,53]). Design strategies meeting the credit requirements were found to reduce the risk of exposure to flooding events by reducing the flooding depth/damage ratio and reducing the risk of urban flooding. By positively affecting the associated environmental determinants of health (urban flooding, population density, water intrusion and exposure to increased levels of microorganism, and community rebuilding after a flooding event), these practices were found to reduce the risk of respiratory disease, flooding-related injury and mortality, disruption to public services, population displacement, and psychological harm to survivors. The co-benefits to built environment outcomes were identified as mitigating flooding severity and funneling rebuilding resources to enhance community cohesion.

Endangered species habitat queried “Biodiversity” and “Urban Health” (seven citations were returned, four of which were relevant to the inquiry [54,55,56,57]). Design strategies meeting the credit requirements were found to reduce the risk of exposure to flooding events by not contributing to urban flooding events. By positively affecting the associated environmental determinants of health (exposure to biodiversity in urban environments, water quality, and reduced exposure to mosquito vectors), these practices were found to improve mental health and wellbeing and to reduce the risk of waterborne disease, respiratory disease, and malaria. More recent studies expand the list of relevant mosquito-borne diseases to include the recent outbreak in the Americas of the Zika virus [112,113]. The co-benefits to built environment outcomes were identified as mitigating the severity of flooding and designing the built environment to reduce mosquito harborage.

Wetlands queried “Wetlands” and “Climate Change” (52 citations were returned, six of which were relevant to the inquiry [58,59,60,61,62,63]). Design strategies meeting the credit requirements were found to reduce the risk of exposure to flooding events by not contributing to the drought/flooding cycle, urban flooding, or coastal flooding/sea level rise. By positively affecting the associated environmental determinants of health (wetland restoration and maintenance, avoided development in low-lying areas near wetlands, water quality, and reduced exposure to mosquito vectors), these practices were found to reduce the risk of waterborne disease, respiratory disease, malaria, and population displacement. More recent studies expand the list of relevant mosquito-borne diseases to include the recent outbreak in the Americas of the Zika virus [112,113]. The co-benefits to built environment outcomes were identified as increasing wildlife habitat, protection from flooding, carbon storage, reducing erosion, and protecting stormwater infrastructure.

Water bodies queried “Cities”, “Climate Change”, “Facility Design and Construction”, “Fresh Water”, “Oceans and Seas”, and “Urban Health” (11 citations were returned, nine of which were relevant to the inquiry [60,64,65,66,67,68,69,70,71]). Design strategies meeting the credit requirements were found to reduce the risk of exposure to flooding events by protecting biodiversity, reducing the burden of stormwater on the local wastewater system, not contributing to coastal flooding and sea level rise, and encouraging responsible land use policies. By positively affecting the associated environmental determinants of health (water quality and exposure to biodiversity in the urban environment), these practices were found to reduce the risk of flooding-related injury and mortality, waterborne disease, water scarcity, disruption to public services, and population displacement. They were also associated with improved mental health and wellbeing. The co-benefits to built environment outcomes were identified as increasing water efficiency; increasing the use of onsite stormwater capture, treatment, and storage; increasing onsite wastewater treatment; contributing to recharging local aquifers; reducing groundwater depletion and subsidence; and, reducing the risk of erosion.

Parkland queried “Biodiversity”, “Conservation of Natural Resources”, and “Facility Design and Construction” (four citations were returned, three of which were relevant to the inquiry [72,73,74]). Design strategies meeting the credit requirements were found to reduce the risk of exposure to flooding events by influencing land use choices. By positively affecting the associated environmental determinants of health (habitat fragmentation and water security), these practices were found to reduce the risk of waterborne disease and interaction between wildlife and humans. The co-benefits to built environment outcomes were identified as clustering development, increasing native vegetation and pervious surface, and encouraging onsite stormwater filtration and storage.

Sustainable Sites Credit 4.1: Alternative Transportation—Public Transportation Access requires projects to locate buildings on sites near public transit stops (Table 3). The literature review queried “Disasters” and “Vulnerable Populations” (149 citations were returned, 11 of which were relevant to the inquiry [75,76,77,78,79,80,82,83,84,85,86]). Additionally, 12 references listed in Farr Associates (2008) [32] were also reviewed, three of which were relevant [81,87,88]). Design strategies meeting the credit requirements were found to have the potential to reduce the risk of exposure to flooding events if ready access to public transit was integrated into municipal evacuation plans, particularly for vulnerable populations such as low income populations and the elderly. Having access to multiple modes of transportation can also reduce stress during and after events. By positively affecting the associated environmental determinants of health (walkability and physical and financial access to multiple modes of transportation during an evacuation—particularly for vulnerable populations), these practices were found to reduce the risk of increased morbidity and mortality during and after flooding events. They can also reduce risk factors for obesity, a determinant of flooding vulnerability due to its tendency to limit mobility [85,114,115,116]. The co-benefits to built environment outcomes were identified as a high mix of land uses, active community design, and improved access to multiple modes of transportation—particularly for vulnerable populations.

Sustainable Sites Credit 4.4: Alternative Transportation—Parking Capacity requires projects to provide preferred parking or dedicated drop-off areas for carpools or to provide fewer total parking spaces (Table 3). No evidence supported providing fewer parking spaces. The results related to carpools duplicated the MeSH queries for Sustainable Sites Credit 4.1.

Sustainable Sites Credit 5.1: Site Development—Protect or Restore Habitat requires projects to limit habitat disturbance during construction or restore habitat on site (Table 3). The literature review queried “Floods”, “Climate Change”, and “Environmental Design” (55 citations were returned, 11 of which were relevant to the inquiry [50,65,89,90,91,92,93,94,95,96,97]). Design strategies meeting the credit requirements were found to reduce the risk of exposure to flooding events by reducing the impact of the drought/flooding cycle, urban flooding, coastal flooding, and sea level rise. By positively affecting the associated environmental determinants of health (percentage pervious cover in neighborhoods with vulnerable populations, water quality, and habitat loss), these practices were found to reduce the risk of flooding-related injury or mortality, waterborne disease, disruption to public services, population displacement, exposure to repeated flooding, combined sewer overflows, mental health problems, chemical toxins, and physical hazards. The co-benefit to built environment outcomes was identified as reducing the risk of property damage due to flooding.

Sustainable Sites Credit 5.2: Site Development—Maximize Open Space requires projects to increase vegetated open space (Table 3). The literature review queried “Floods”, “Climate Change”, “Environment Design”, and “Wetlands” (107 citations were returned, 17 of which were relevant to the inquiry [50,58,59,60,61,62,63,65,89,90,91,92,93,94,95,96,97]). Design strategies meeting the credit requirements were found to reduce the risk of exposure to flooding events by reducing the impact of the drought/flooding cycle, urban flooding, coastal flooding, and sea level rise. By positively affecting the associated environmental determinants of health (percentage pervious cover in neighborhoods with vulnerable populations, water quality, and habitat loss), these practices were found to reduce the risk of flooding-related injury or mortality, waterborne disease, malaria, disruption to public services, population displacement, exposure to repeated flooding, combined sewer overflows, mental health problems, chemical toxins, and physical hazards. More recent studies expand the list of relevant mosquito-borne diseases to include the recent outbreak in the Americas of the Zika virus [112,113]. The co-benefits to built environment outcomes were identified as reducing the risk of property damage due to flooding and increasing wildlife habitat.

Sustainable Sites Credit 6.1: Stormwater Design—Quantity Control requires projects to design the site to reduce the post-development peak quantity of stormwater discharge after heavy precipitation events (Table 3). The literature review queried “Floods”, “Climate Change”, “Environment Design”, and “Urbanization” (112 citations were returned, 18 of which were relevant to the inquiry [50,68,89,90,92,93,94,95,96,97,98,99,100,101,102,103,104,105]). Design strategies meeting the credit requirements were found to reduce the risk of exposure to flooding events by reducing the impact of the drought/flooding cycle, urban flooding, coastal flooding, and sea level rise. By positively affecting the associated environmental determinants of health (river basin retention capacity, percentage pervious cover in neighborhoods with vulnerable populations, and water quality), these practices were found to reduce the risk of flooding-related injury or mortality, waterborne disease, exposure to repeated flooding, combined sewer overflows, chemical toxins, and physical hazards. The co-benefit to built environment outcomes was identified as reducing the risk of property damage due to flooding.

Sustainable Sites Credit 6.2: Stormwater Design—Quality Control requires projects to design the site to remove pollution from stormwater runoff (Table 3). The literature review queried “Floods”, “Climate Change”, “Environment Design”, “Urbanization”, and “Wetlands” (164 citations were returned, 21 of which were relevant to the inquiry [58,59,60,62,63,65,66,68,90,91,92,94,96,97,98,100,102,106,107,108,109]). Design strategies meeting the credit requirements were found to reduce the risk of exposure to flooding events by reducing the quantity of compromised water and wastewater leaving the site and by reducing the impact of the drought/flooding cycle, urban flooding, coastal flooding, and sea level rise. By positively affecting the associated environmental determinants of health (percentage pervious cover in neighborhoods with vulnerable populations, water quality, habitat loss, and wetland restoration and maintenance), these practices were found to reduce the risk of flooding-related injury or mortality, waterborne disease, malaria, disruption to public services, population displacement, exposure to repeated flooding, combined sewer overflows, mental health problems, chemical toxins, and physical hazards. More recent studies expand the list of relevant mosquito-borne diseases to include the recent outbreak in the Americas of the Zika virus [112,113]. The co-benefits to built environment outcomes were identified as reducing the risk of property damage due to flooding and increasing wildlife habitat.

Sustainable Sites Credit 7.1: Heat Island Effect—Non-roof requires projects to shade impervious surfaces on-site, install light-colored or pervious hardscape, or install covered parking areas (Table 3). No evidence supported the third option. The literature review queried “Floods”, “Climate Change”, “Environment Design”, and “Urbanization” (112 citations were returned, 11 of which were relevant to the inquiry [50,68,89,90,91,92,93,94,96,97,102]). Design strategies meeting the credit requirements were found to reduce the risk of exposure to flooding events by reducing the quantity of compromised water and wastewater leaving the site and by reducing the impact of urban flooding. By positively affecting the associated environmental determinants of health (percentage pervious cover in neighborhoods with vulnerable populations, water quality, habitat loss, wetland restoration and maintenance), these practices were found to reduce the risk of flooding-related injury or mortality, waterborne disease, malaria, disruption to public services, population displacement, exposure to repeated flooding, combined sewer overflows, mental health problems, chemical toxins, and physical hazards. More recent studies expand the list of relevant mosquito-borne diseases to include the recent outbreak in the Americas of the Zika virus [112,113]. The co-benefits to built environment outcomes were identified as reducing the risk of property damage due to flooding and increasing wildlife habitat.

Sustainable Sites Credit 7.2: Heat Island Effect—Roof requires projects to install light colored roofs or vegetated roofs on the building (Table 3). No evidence supported the first option. The literature review queried “Climate Change” and “Urbanization” (57 citations were returned, three of which were relevant to the inquiry [68,100,103]). Design strategies meeting the credit requirements were found to reduce the risk of exposure to flooding events by reducing the site’s burden on the municipal wastewater system from stormwater runoff and reducing the impact of urban flooding. By positively affecting the associated environmental determinants of health (percentage pervious cover in neighborhoods with vulnerable populations, water quality, and habitat loss), these practices were found to reduce the risk of flooding-related injury or mortality and waterborne disease. The co-benefits to built environment outcomes were identified as reducing the risk of property damage due to flooding and increasing wildlife habitat.

Water Efficiency Credit 1: Water Efficient Landscaping requires projects to reduce potable water consumption for irrigation (Table 3). The literature review queried “Biodiversity”, “Cities”, “Climate Change”, “Conservation of Natural Resources”, “Facility Design and Construction”, “Fresh Water”, and “Urban Health” (15 citations were returned, 11 of which were relevant to the inquiry [54,55,56,65,66,67,68,72,73,74,110]). Design strategies meeting the credit requirements were found to reduce the risk of exposure to flooding events by increasing biodiversity and by reducing the impact of urban flooding, coastal flooding, and sea level rise. By positively affecting the associated environmental determinants of health (biodiversity in urban environments, habitat fragmentation, and water security), these practices were found to reduce the risk of waterborne disease and interaction between wildlife and humans. The co-benefits to built environment outcomes were identified as increasing onsite wastewater and stormwater treatment and storage, clustering development, and increasing native vegetation and pervious surfaces.

Water Efficiency Credit 2: Innovative Wastewater Technologies requires projects to reduce potable water use for building sewage conveyance (Table 3). The literature review queried “Cities”, “Climate Change”, “Environment Design”, “Fresh Water”, “Facility Design and Construction”, “Urban Health”, and “Water Pollution” (six citations were returned, all of which were relevant to the inquiry [64,65,66,67,68,111]). Design strategies meeting the credit requirements were found to reduce the risk of exposure to flooding events by reducing the site’s burden on the municipal wastewater system from stormwater runoff and reducing the impact of urban flooding, coastal flooding, and sea level rise. By positively affecting the associated environmental determinants of health (water quality and biodiversity in urban environments), these practices were found to reduce the risk of flooding-related illness, waterborne disease, and water scarcity. They were also associated with improved mental health and wellbeing. The co-benefits to built environment outcomes were identified as increasing water efficiency and onsite water capture and treatment practices, recharging local aquifers, and reducing groundwater depression and subsidence.

Water Efficiency Credit 3: Water Use Reduction requires projects to reduce potable water consumption inside the building (Table 3). The literature review queried “Cities”, “Climate Change”, and “Fresh Water” (four citations were returned, all of which were relevant to the inquiry [65,66,67,68].) Design strategies meeting the credit requirements were found to reduce the risk of exposure to flooding events by protecting biodiversity and reducing the impact of urban flooding, coastal flooding, and sea level rise. By positively affecting the associated environmental determinants of health (water quality and biodiversity in urban environments), these practices were found to reduce the risk of flooding-related illness, waterborne disease, and water scarcity. They were also associated with improved mental health and wellbeing. The co-benefits to built environment outcomes were identified as increasing water efficiency and onsite water capture and treatment practices, recharging local aquifers, and reducing groundwater depression and subsidence.

In summary, the literature review revealed a number of common themes linking green building strategies with protective features associated with urban flooding. The dominant environmental determinants of health were identified as: exposure to biodiversity in urban environments; habitat fragmentation and loss; percentage pervious cover in neighborhoods with vulnerable populations; and, water quality. Co-benefits to health included reduced risk of flooding-related injury and mortality, waterborne disease, mental illness, and interface between humans and wildlife. The most salient co-benefits to the built environment could be characterized as a combination of dense, mixed-use developments with access to multiple modes of transportation interspersed with increased wildlife habitat and arable land. This combination can both reduce the risk of property damage due to flooding and increase the local population’s resilience to floods when they do occur. Overall, the results from the flooding resilience literature review were more diverse than the review of extreme heat resilience (see companion article, “Associations between green building design strategies and community resilience to extreme heat events: a review of the evidence”). The credits included in the flooding resilience review also favored outdoor strategies, such as site selection, parking layouts, and landscape design; whereas, the credits included in the extreme heat literature review included a more balanced representation of green building practices influencing the design of the site, the building footprint and massing, and the interior.

## 4. Discussion

### 4.1. Opportunities to Strengthen the LEED Rating Systems

The results of the literature review identified 12 LEED credits that, in addition to their stated intent, offer the opportunity to reduce negative health outcomes when building occupants are exposed to a flooding event. However, given the gravity of public health concerns associated with extreme weather events linked to climate change, it may be time for LEED to add a bundle of emergency preparedness credit to supplement the base rating system. The first step in this direction is the LEED pilot credits on resilient design. This is a proposed suite of three credits that provide the opportunity for project teams to: (1) perform a hazard assessment to identify the three natural hazards that pose the highest risk for the site; (2) incorporate design elements to protect building occupants from those hazards; and, (3) incorporate design elements that will allow the building to continue functioning in the event of disruption to key utilities (i.e., design for passive survivability) [117]. However, it should be noted that the current draft of the pilot credits does not address vulnerable populations outside of emergency preparedness planning. The credits focus on enhancing the resilience of infrastructure. Additional work would be required to overlay environmental determinants of health and public health outcomes, similar to the assessment outlined in this article.

The results of this literature review also offer important opportunities for collaboration among the public health, climate change, civil society, and green building sectors in a number of areas, including: flooding resilience policies, community planning, and transportation planning.

### 4.2. Flooding Resilience Policies

This literature review was conducted as part of a project in Travis County, Texas, (where the capital, Austin, is located) whose goal was to provide a decision-making tool for local policymakers, so that funds earmarked for climate change-related interventions would target the most vulnerable populations in the city [118]. The project developed a pair of climate change vulnerability maps at the neighborhood level assessing the combined socio-demographic and environmental vulnerability of Travis County residents to extreme heat and flooding events, respectively. Based on the results of the vulnerability maps, published elsewhere [118,119], the project team identified three local policies that might consider prioritizing neighborhoods with high vulnerability for early interventions: the City of Austin sidewalk master plan, incentives encouraging installation of vegetated roofs, and the water quality program designed to remediate impaired streams. The results of this literature review further suggest green building strategies that the City might consider incentivizing or requiring in highly vulnerable neighborhoods.

The U.S. Federal Emergency Management Agency (FEMA) offers another example of ways flooding resilience policies could be strengthened through input from research linking climate change with health outcomes and green building strategies. The 2014–2018 FEMA Strategic Plan includes an objective (4.2) to “incentivize and facilitate investment” in buildings and infrastructure that will reduce current and future economic losses related to disasters, particularly flooding-related risks [120]. Two policies in particular, Sections 404 and 406 of the Stafford Act, provide funding following disaster declarations to fortify buildings and infrastructure from potential losses related to future disasters. Other programs, such as the Pre-Disaster Mitigation Grant Program (http://www.fema.gov/pre-disaster-mitigation-grant-program), fund policies and projects aimed at mitigating risks associated with future disasters. However, these policies currently do not mention social vulnerabilities, underlying health risks, or the environmental and economic co-benefits associated with relevant green building practices. The literature review outlined in this article could inform the most efficient use of available funding by identifying the strongest co-benefits linking a proposed project or policy with regional health-related climate vulnerabilities and the most protective design strategies as supported by the evidence.

At the international level, United Nations Sustainable Development Goal 6: Clean water and sanitation [121] could make use of the research outlined in this literature review to highlight the role that climate change plays in increasing the risk of exposure to contaminated flood waters in vulnerable urban areas.

### 4.3. Community Planning

Perhaps one of the most comprehensive recent attempts to integrate flooding resilience into community planning and building policies is the New York City plan, A Stronger, More Resilient New York [122], which was developed in response to the damage caused by Hurricane Sandy in 2012. This plan and the recommendations of the related Building Resilience Task Force [123] have focused on reducing the risk of wind damage and raising the first floor of occupied space and building equipment above the 100-year flood plain (which FEMA expanded post-Sandy to reflect updated approximations of flood risk) [122]. Code recommendations also call for encouraging on-site backup power using renewable sources such as cogeneration and solar panels. The Building Resilience Task Force recommendations also encourage passive survivability (i.e., strategies that allow occupants to continue to use a building when the power and water are disconnected [117])—for example, operable windows, water fixtures that can function when the power is out, and emergency lighting that functions for an extended period of time without electrical power [123]. The results of the literature review outlined in this article could be used as an evidence base by similar initiatives in the future. While several of the green building strategies supported by the literature review were incorporated into the final recommendations offered by the Building Resilience Task Force—such as, capturing stormwater (Sustainable Sites Credit 6.1: Stormwater Design—Quantity Control), reducing the urban heat island effect (Sustainable Sites Credit 7.2: Heat Island Effect—Roof), and water efficiency measures (Water Efficiency Credit 2: Innovative Wastewater Technologies and Water Efficiency Credit 3: Water Use Reduction)—a number of them were not. Furthermore, the results of the literature review could be used to harmonize existing green building regulations with new code requirements focused on climate change resilience. This approach could reduce the confusion associated with navigating overlapping building codes, and thereby reducing construction cost and increasing compliance rates.

### 4.4. Transportation Planning

One of the key findings of the literature review is the link between flooding vulnerability and access to transportation to evacuate vulnerable areas before, during, and after flooding events. Cambridge, MA’s climate change vulnerability assessment reflects these results, predicting that several subway and commuter rail hubs as well as three key bridges crossing the Charles River will represent priority vulnerabilities for the City by 2030 [124]. The results of this literature review could be used as an evidence base in similar assessments in the future. They also open the door to coordination between community-scale transportation planning and site- and neighborhood-scale green building projects, an opportunity for synergies that is often overlooked by both parties.

Furthermore, they could be used to fill information gaps in tools such as the Transportation and Health Tool (https://www.transportation.gov/transportation-health-tool)—a set of transportation and public health indicators jointly developed by the U.S. Department of Transportation and the U.S. Centers for Disease Control and Prevention—which currently does not include the protective role that access to multiple modes of transportation can play for vulnerable populations during flooding events.

### 4.5. Study Strengths and Limitations

This study filled important gaps in the literature linking specific green building strategies with the environmental determinants of health associated with urban flooding. It also highlights opportunities for green building practices to protect both population health and the built environment from the worst ravages of urban flooding events. Because the study brought together datasets from two disciplines that rarely collaborate (green building and public health), its results are widely applicable to both of those sectors, as well as their traditional partners, such as: real estate, civil society, law enforcement, emergency preparedness and response, etc. It also demonstrates the value of cross-sectorial collaboration, both in the academic and research communities and in the field.

The study’s limitations include the methodology’s inability to measure the strengths of associations between LEED credits and health outcomes, its emphasis on voluntary credits in the LEED rating system and exclusion of prerequisites from review, and the small number of studies linking health with built environment interventions. A great deal of additional research is needed in this area to quantify the magnitude of health benefits associated with specific green building design strategies. Pre-/post-intervention studies and predictive modeling are two methods that could be employed to begin filling this gap in knowledge. Future studies should expand this review to include other climatic events, such as drought, wildfires, hurricanes, and vector-borne diseases.

## 5. Conclusions

Flooding poses a serious and growing threat to public health under a changing climate. Given the interplay between the built environment and flooding-related injuries and deaths, architectural design has the potential to act as a protective public health intervention.

The systematic literature review outlined in this article found evidence that certain green building strategies have the potential to reduce the risk of negative health outcomes following exposure to flooding. Water quality, habitat loss and fragmentation, exposure to biodiversity, and percentage pervious cover in neighborhoods with vulnerable populations were identified as key environmental determinants of health linking green building strategies and flooding events. The public health co-benefits of these strategies include reducing the risk of waterborne diseases, flood-related morbidity and mortality, psychological harm, and interface between humans and wildlife.

The results of this literature review demonstrate the value that public health evidence can bring to collaborations among the public health, climate change, civil society, and green building sectors on topics such as flooding resilience, community planning, and transportation planning. The LEED credits called out in this literature review could be implemented proactively by property owners and architectural design teams to enhance the resilience of a site located in an area that is highly vulnerable to urban flooding events. It could also provide an evidence base for public health, community planning, and zoning decisions aimed at increasing community resilience to climate change.

## Figures and Tables

**Table 1:** Health-related prerequisites and credits in LEED version 2009 commercial rating systems by section.

Sustainable Sites	Water Efficiency	Energy & Atmosphere	Materials & Resources	Indoor Environmental Quality	Innovation
***Prerequisites***	***Prerequisites***	***Prerequisites***	***Prerequisites***	***Prerequisites***	***Prerequisites***
			HC-Avoid Mercury	Environmental Tobacco Smoke Control	
				HC-Acoustics	
***Voluntary Credits***	***Voluntary Credits***	***Voluntary Credits***	***Voluntary Credits***	***Voluntary Credits***	***Voluntary Credits***
Brownfield Mitigation	Wastewater Reduction	HC-Emissions Limits	HC-Avoid PBTs	Ventilation	
HC-Places of Respite				Thermal Comfort	
HC-Exterior Access				Low-Emitting Materials	
				Construction Indoor Air Quality	
				Chemical & Pollutant Source Control	
				Daylighting, Views	
				Schools-Mold Prevention	
				HC/Schools-Acoustics	

Abbreviations: HC: LEED for Healthcare; Schools: LEED for Schools.

**Table 2:** LEED credits included in flooding resilience literature review.

LEED Credit Title *Description*
* Sustainable Sites Credit 1: Site Selection *Avoid building on: prime farmland; land in 100-year flood plain; endangered species habitat; land within 100 feet of wetlands or 50 feet of water bodies; park land.*
Sustainable Sites Credit 4.1: Alternative Transportation—Public Transportation Access *Locate project near bus/rail lines.*
Sustainable Sites Credit 4.4: Alternative Transportation—Parking Capacity *Provide preferred parking areas for carpools/vanpools.*
* Sustainable Sites Credit 5.1: Site Development—Protect or Restore Habitat *Limit disturbance of habitat on greenfield sites. Restore habitat on previously developed habitat.*
* Sustainable Sites Credit 5.2: Site Development—Maximize Open Space *Increase vegetated open space.*
* Sustainable Sites Credit 6.1: Stormwater Design—Quantity Control *Reduce the volume of stormwater that leaves the site after heavy precipitation events.*
* Sustainable Sites Credit 6.2: Stormwater Design—Quality Control *Clean stormwater of total suspended solids.*
* Sustainable Sites Credit 7.1: Heat Island Effect—Non-roof *Install light colored and pervious paving (i.e., roads, sidewalks, parking lots, etc.) or place at least 1/2 of all parking spaces under cover.*
* Sustainable Sites Credit 7.2: Heat Island Effect—Roof *Install light colored or vegetated roofs.*
Water Efficiency Credit 1: Water Efficient Landscaping *Reduce potable water use for irrigation by 50% or 100%.*
Water Efficiency Credit 2: Innovative Wastewater Technologies *Reduce potable water use for sewage conveyance.*
Water Efficiency Credit 3: Water Use Reduction *Reduce potable water use for interior fixtures (i.e., toilets, lavatories, showers, etc.).*

Note: * Evidence of contribution to both heat resilience and flooding resilience. Source: LEED Reference Guide for Green Building Design and Construction [26].

**Table 3:** Association between LEED credits and community resilience to urban flooding events: A review of the evidence.

LEED Credit	Requirements	MeSH Query Terms	Relevant Citations (Total Queried in PubMed and Farr Associates (2008) [32])	How Strategy Impacts Risk of Exposure	Environmental Determinants of Health	Co-Benefits to Public Health Outcomes	Co-Benefits to Built Environment Outcomes
Sustainable Sites Credit 1: Site Selection	Avoid development in or adjacent to the following areas						
	Prime farmland [44,45,46,47,48]	Agriculture Climate Change Facility Design and Construction Urbanization	5 (17)	Habitat fragmentation Land use changes Sprawl development	Access to opportunities to exercise. Dependence on automobiles. Food and nutrition security. Food safety. Habitat fragmentation.	Reduced risk of flooding-related injury; under- and mal-nutrition; infectious disease; interface between wildlife and humans.	Reduced development in areas without services. Increased access to local, productive agricultural land.
	Floodplain [49,50,51,52,53]	Floods Facility Design and Construction	5 (7)	Flooding depth/damage ratio Urban flooding	Urban flooding. Population density. Water intrusion and exposure to increased levels of microorganism. Community rebuilding after a flooding event.	Reduced risk of respiratory disease. Reduced flooding-related injury and mortality. Reduced risk of disruption to public services. Reduced risk of population displacement. Reduced psychological harm to survivors.	Severity of flooding mitigated. Funnel rebuilding resources to enhance community cohesion.
	Endangered Species Habitat [54,55,56,57]	Biodiversity Urban Health	4 (7)	Urban flooding	Exposure to biodiversity in urban environments. Water quality. Exposure to mosquito vectors.	Improved mental health and wellbeing. Reduced risk of waterborne disease, respiratory disease, and malaria.	Severity of flooding mitigated. Design built environment to not harbor mosquitoes.
	Wetlands [58,59,60,61,62,63]	Wetlands Climate Change	6 (52)	Drought/flooding cycle Urban flooding Coastal flooding/Sea level rise	Wetland restoration and maintenance. Development in low-lying areas near wetlands. Water quality. Exposure to mosquito vectors.	Reduced risk of waterborne disease, respiratory disease, malaria, and population displacement.	Increased wildlife habitat, protection from flooding, carbon storage. Reduced erosion. Protected stormwater infrastructure.
	Water Body [60,64,65,66,67,68,69,70,71]	Cities Climate Change Facility Design and Construction Fresh Water Oceans and Seas Urban Health	9 (11)	Biodiversity Burden on wastewater system from stormwater runoff Coastal flooding/Sea level rise Land use changes	Water quality. Exposure to biodiversity in urban environment.	Reduced risk of flooding-related injury and mortality; waterborne disease; water scarcity; disruption to public services; population displacement. Improved mental health and wellbeing.	Increased water efficiency; onsite stormwater capture, treatment, and storage; onsite wastewater treatment; aquifer recharge. Reduced groundwater depletion; subsidence. Reduce risk of erosion.
	Parkland [72,73,74]	Biodiversity Conservation of Natural Resources Facility Design and Construction	3 (4)	Land use changes	Habitat fragmentation. Water security.	Reduced risk of interface between wildlife and humans; waterborne disease.	Cluster development. Increased native vegetation and pervious surface. Onsite stormwater filtration and storage.
Sustainable Sites Credit 4.1: Alternative Transportation—Public Transportation Access	Locate building on a site near public transit stops [75,76,77,78,79,80,81,82,83,84,85,86,87,88].	Disasters Vulnerable Populations	14 (161)	Ability to evacuate Stress during and after events	Physical and financial access to multiple modes of transportation during an evacuation (particularly to vulnerable populations). Walkability.	Reduced risk factors for obesity (precondition of vulnerability to flooding). Evacuation plan to reduce risk of increased morbidity and mortality among older adults during and after natural disasters.	High mix of land uses. Active community design. Improved access to multiple modes of transportation, particularly for vulnerable populations.
Sustainable Sites Credit 4.4: Alternative Transportation—Parking Capacity	Provide preferred parking or dedicated drop-off areas for carpools. No evidence for reducing total parking capacity [75,76,77,78,79,80,82,83,84,85,86].	Disasters Vulnerable Populations	11 (149)	Ability to evacuate Stress during and after events	Physical and financial access to multiple modes of transportation during an evacuation (particularly to vulnerable populations). Walkability.	Reduced risk factors for obesity (precondition of vulnerability to flooding). Evacuation plan to reduce risk of increased morbidity and mortality among older adults during and after natural disasters.	High mix of land uses. Improved access to multiple modes of transportation, particularly for vulnerable populations.
Sustainable Sites Credit 5.1: Site Development—Protect or Restore Habitat	Limit habitat disturbance during construction or restore habitat [50,65,89,90,91,92,93,94,95,96,97].	Floods Climate Change Environment Design	11 (55)	Drought/flooding cycle Urban flooding Coastal flooding/Sea level rise	Percentage pervious cover in neighborhoods with vulnerable populations. Water quality. Habitat loss.	Reduced risk of flooding-related injury or mortality; waterborne disease; disruption to public services; population displacement; exposure to repeated flooding; combined sewer overflows; mental health problems; chemical toxins and physical hazards.	Reduced risk of property damage due to flooding.
Sustainable Sites Credit 5.2: Site Development—Maximize Open Space	Increase vegetated open space [50,58,59,60,61,62,63,65,89,90,91,92,93,94,95,96,97].	Floods Climate Change Environment Design Wetlands	17 (107)	Drought/flooding cycle Urban flooding Coastal flooding/Sea level rise	Percentage pervious cover in neighborhoods with vulnerable populations. Water quality. Habitat loss.	Reduced risk of flooding-related injury or mortality; waterborne disease; malaria; disruption to public services; population displacement; exposure to repeated flooding; combined sewer overflows; mental health problems; chemical toxins and physical hazards.	Reduced risk of property damage due to flooding. Increase wildlife habitat.
Sustainable Sites Credit 6.1: Stormwater Design—Quantity Control	Design the site to reduce the post-development peak discharge quantity after heavy precipitation events [50,68,89,90,92,93,94,95,96,97,98,99,100,101,102,103,104,105].	Floods Climate Change Environment Design Urbanization	18 (112)	Drought/flooding cycle Urban flooding Coastal flooding/Sea level rise	River basin retention capacity. Percentage pervious cover in neighborhoods with vulnerable populations. Water quality.	Reduced risk of flooding-related injury or mortality; waterborne disease; exposure to repeated flooding; combined sewer overflows; chemical toxins and physical hazards.	Reduced risk of property damage due to flooding.
Sustainable Sites Credit 6.2: Stormwater Design—Quality Control	Design the site to remove pollution from stormwater runoff [58,59,60,62,63,65,66,68,90,91,92,94,96,97,98,100,102,106,107,108,109].	Floods Climate Change Environment Design Urbanization Wetlands	21 (164)	Compromised water and wastewater quality Drought/flooding cycle Urban flooding Coastal flooding/Sea level rise	Percentage pervious cover in neighborhoods with vulnerable populations. Water quality. Habitat loss. Wetland restoration and maintenance.	Reduced risk of flooding-related injury or mortality; waterborne disease; malaria; disruption to public services; population displacement; exposure to repeated flooding; combined sewer overflows; mental health problems; chemical toxins and physical hazards.	Reduced risk of property damage due to flooding. Increased wildlife habitat.
Sustainable Sites Credit 7.1: Heat Island Effect—Non-roof	Shade impervious surfaces on-site, install light-colored or pervious hardscape, or install covered parking [50,68,89,90,91,92,93,94,96,97,102].	Floods Climate Change Environment Design Urbanization	11 (112)	Compromised water and wastewater quality Urban flooding	Percentage pervious cover in neighborhoods with vulnerable populations. Water quality. Habitat loss. Wetland restoration and maintenance.	Reduced risk of flooding-related injury or mortality; waterborne disease; malaria; disruption to public services; population displacement; exposure to repeated flooding; combined sewer overflows; mental health problems; chemical toxins and physical hazards.	Reduced risk of property damage due to flooding. Increased wildlife habitat.
Sustainable Sites Credit 7.2: Heat Island Effect—Roof	Install light colored roof or vegetated roof. [68,100,103]	Climate Change Urbanization	3 (57)	Burden on wastewater system from stormwater runoff Urban flooding	Percentage pervious cover in neighborhoods with vulnerable populations. Water quality. Habitat loss.	Reduced risk of flooding-related injury or mortality; waterborne disease.	Reduced risk of property damage due to flooding. Increased wildlife habitat.
Water Efficiency Credit 1: Water Efficient Landscaping	Reduce potable water consumption for irrigation [54,55,56,65,66,67,68,72,73,74,110].	Biodiversity Cities Climate Change Conservation of Natural Resources Facility Design and Construction Fresh Water Urban Health	11 (15)	Biodiversity Urban flooding Coastal flooding/Sea level rise	Exposure to biodiversity in urban environments. Habitat fragmentation. Water security.	Reduced risk of waterborne disease; interface between wildlife and humans.	Increased onsite wastewater and stormwater treatment and storage. Cluster development. Increased native vegetation and pervious surfaces.
Water Efficiency Credit 2: Innovative Wastewater Technologies	Reduce potable water use for building sewage conveyance [64,65,66,67,68,111].	Cities Climate Change Environment Design Fresh Water Facility Design and Construction Urban Health Water Pollution	6 (6)	Burden on wastewater system and waterways from stormwater runoff Urban flooding Coastal flooding/Sea level rise	Water quality. Exposure to biodiversity in urban environments.	Reduced risk of flooding-related illness, waterborne disease, water scarcity; Improved mental health and wellbeing.	Increased water efficiency; onsite water capture and treatment; recharge aquifer. Reduced groundwater depression; subsidence.
Water Efficiency Credit 3: Water Use Reduction	Reduce potable water consumption inside the building [65,66,67,68].	Cities Climate Change Fresh Water	4 (4)	Biodiversity Urban flooding Coastal flooding/Sea level rise	Water Quality. Exposure to biodiversity in urban environments.	Reduced risk of flooding-related illness, waterborne disease, water scarcity; Improved mental health and wellbeing.	Increased water efficiency, onsite water capture and treatment, recharge aquifer. Reduced groundwater depression, subsidence.

## Supplementary Materials

The following are available online at www.mdpi.com/1660-4601/14/12/1519/s1, Table S1: MeSH Terms queried by LEED credit.
