# Supplementary material for: Health Co-Benefits of Green Building Design Strategies and Community Resilience to Urban Flooding: A Systematic Review of the Evidence
Source: Int J Environ Res Public Health. 2017 Dec 6;14(12):1519. doi: 10.3390/ijerph14121519 (PMC5750937; doi:10.3390/ijerph14121519)
Supplement: Supplementary file 1 [file ijerph-14-01519-s001.docx]

**Table S1:** MeSH Terms queried by LEED credit.

| **LEED Credit Title** *Requirement(s): “MeSH Terms”* |
| --- |
| Sustainable Sites Credit 1: Site Selection  *Prime farmland: "Agriculture” AND "Facility Design and Construction" AND "Climate Change";  "Agriculture" AND "Urbanization” AND "Climate Change”. Land in 100-year flood plain: "Floods" AND "Facility Design and Construction". Endangered species habitat: "Biodiversity" AND "Urban Health".  Land within 100 feet of wetlands or 50 feet of water bodies: "Wetlands" AND "Climate Change";  "Facility Design and Construction” AND "Urban Health" AND "Fresh Water";  "Climate Change" AND "Fresh Water" AND "Cities"; "Climate Change" AND "Oceans and Seas". Park land:* *"Conservation of Natural Resources" AND "Biodiversity" AND "Facility Design and Construction”.* |
| Sustainable Sites Credit 4.1: Alternative Transportation—Public Transportation Access  *Locate project near bus/rail lines: "Transportation” AND "Floods" AND "Climate Change";*  *"Disasters" AND "Vulnerable Populations”.* |
| Sustainable Sites Credit 4.4: Alternative Transportation—Parking Capacity  *Provide preferred parking areas for carpools/vanpools: "Transportation” AND "Floods" AND "Climate Change";*  *"Disasters" AND "Vulnerable Populations”.* |
| Sustainable Sites Credit 5.1: Site Development—Protect or Restore Habitat  *Limit disturbance of habitat on greenfield sites: "Floods" AND "Environment Design”. Restore habitat on previously developed habitat:* *"Floods" AND "Climate Change".* |
| Sustainable Sites Credit 5.2: Site Development—Maximize Open Space  *Increase vegetated open space: "Floods" AND "Environment Design”; "Floods" AND "Climate Change";  "Wetlands” AND "Climate Change".* |
| Sustainable Sites Credit 6.1: Stormwater Design—Quantity Control  *Reduce the volume of stormwater that leaves the site after heavy precipitation events: “Floods" AND "Environment Design"; "Floods" AND "Climate Change"; "Urbanization" AND "Climate Change".* |
| Sustainable Sites Credit 6.2: Stormwater Design—Quality Control  *Clean stormwater of total suspended solids: "Floods" AND "Climate Change"; "Wetlands" AND "Climate Change".* |
| Sustainable Sites Credit 7.1: Heat Island Effect—Nonroof  *Install light colored and pervious paving (i.e., roads, sidewalks, parking lots, etc): "Urbanization" AND "Climate Change"; “Floods” AND “Environment Design”; “Floods” AND “Climate Change”. Place at least 1/2 of all parking spaces under cover: No queries relevant to flooding.* |
| Sustainable Sites Credit 7.2: Heat Island Effect—Roof  *Install light colored or vegetated roofs: "Urbanization" AND "Climate Change”.* |
| Water Efficiency Credit 1: Water Efficient Landscaping  *Reduce potable water use for irrigation by 50% or 100%: "Biodiversity" AND "Urban Health";  "Climate Change" AND "Fresh Water" AND "Cities";  "Conservation of Natural Resources" AND "Biodiversity" AND "Facility Design and Construction".* |
| Water Efficiency Credit 2: Innovative Wastewater Technologies  *Reduce potable water use for sewage conveyance : "Facility Design and Construction" AND "Urban Health" AND "Fresh Water";*  *"Water Pollution” AND "Climate Change” AND "Environment Design"; "Climate Change" AND "Fresh Water" AND "Cities".* |
| Water Efficiency Credit 3: Water Use Reduction  *Reduce potable water use for interior fixtures (i.e., toilets, lavatories, showers, etc.):  "Climate Change" AND "Fresh Water" AND "Cities".* |
